# Alternative Lys277 cleavage modulates tissue plasminogen activator processing and fibrinolysis

**DOI:** 10.1016/j.rpth.2026.107008

**Published:** 2026-10-12

**Authors:** Simon Lebatard, Laurent Lebouvier, Yannick Hommet, Anne Ciavatti Belpois, Sacha Ahuir, Viviane A. Bastos, Solenne Chardonnet, Donna Pinheiro, Robert L. Medcalf, Sara Martinez de Lizarrondo, Richard Macrez, Denis Vivien

**Affiliations:** 1 Physiology and Imaging of Neurological Disorders Unité Mixte de Recherche - Santé (PhIND UMR-S 1237), Institut national de la santé et de la recherche médicale (Inserm), Brain, Blood and Memory @ Caen Normandie Institute (BBM@C), University of Caen Normandie, Caen, France; 2 Unité de Service Production et Analyse de données en Sciences de la vie et en Santé (US PASS), Plateforme Post-Génomique de la Pitié-Salpêtrière, Sorbonne Université, Paris, France; 3 Molecular Neurotrauma and Haemostasis Laboratory, Australian Centre of Blood Diseases, School of Translational Medicine, Monash University, Melbourne, Victoria, Australia; 4 Department of Emergency Medicine, Caen Normandie University Hospital, Caen, France; 5 Department of Clinical Research, Caen Normandie University Hospital, Caen, France

**Keywords:** fibrinolysis, phylogeny, protein isoforms, thrombolysis, tissue plasminogen activator

## Abstract

**Background:**

Tissue plasminogen activator (tPA) is secreted as a single-chain enzyme and converted to its canonical 2-chain form by cleavage at Arg275. Whether the adjacent Lys277 residue contributes to tPA activation and fibrinolytic function remains unclear.

**Objectives:**

To determine whether Lys277 can serve as an alternative tPA cleavage site and whether Lys277-dependent processing is associated with altered fibrinolytic activity.

**Methods:**

We combined phylogenetic analysis of PLAT sequences from 645 gnathostome species, engineered human tPA mutants targeting Arg275 and/or Lys277, plasmin- and plasma-cleavage assays, Edman sequencing, amidolytic and fibrin-plasminogen zymography, plasma clot lysis assays, and rotational thromboelastometry.

**Results:**

Arg275 was highly conserved across species, whereas species with Arg275 substitutions almost invariably retained Lys277; snakes contained only the Lys277 site. Plasmin cleaved wild-type tPA at Arg275-Ile276, whereas Arg275-mutant tPA was cleaved at Lys277-Gly278; mutation of both residues prevented 2-chain tPA formation. Compared with wild-type single-chain tPA, Arg275 single mutants reduced 50% lysis of fibrin-rich plasma clots by 1.9- to 2.4-fold and accelerated fibrinolysis of blood clots. Human plasma generated tPA heavy-chain fragments from wild-type and Arg275-mutant tPA, but not from the Arg275/Lys277 double mutant, indicating cleavage at both sites under plasma conditions. Preparations enriched in Lys277-cleaved tPA showed greater blood clot lysis than those enriched in Arg275-cleaved tPA, with corrected fibrinolysis up to 2.8-fold higher.

**Conclusion:**

These data support Lys277 as an evolutionarily conserved alternative tPA cleavage site that can generate a 2-chain isoform with distinct fibrinolytic properties *in vitro* and *ex vivo*.

## Essentials

• Tissue plasminogen activator (tPA) supports fibrinolysis through single- and two-chain forms with distinct functional properties.
• Phylogenetic analysis, site-directed mutagenesis, and biochemical and human clot lysis assays were combined to investigate alternative tPA cleavage.
• Plasmin cleaves R275-mutant tPA at Lys277 in vitro, generating a distinct two-chain form, tc(K277)-tPA.
• Variants retaining Lys277 cleavage exhibit enhanced clot lysis in vitro, supporting further investigation of this site for thrombolytic development.

## Graphical abstract

**Figure:**
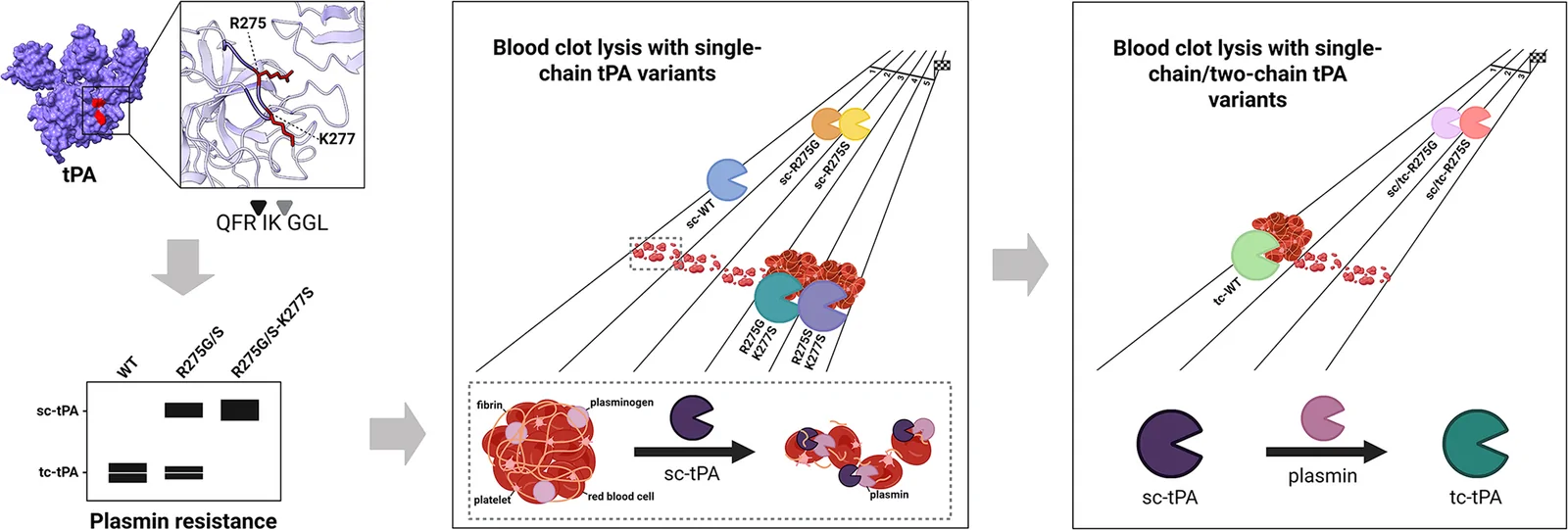

## 1 Introduction

Tissue plasminogen activator (tPA) is a serine protease secreted into the bloodstream by endothelial cells and is best known for promoting clot lysis through vascular fibrinolysis by converting fibrin-bound plasminogen to plasmin [1]. Cleavage at the Arg275-Ile276 peptide bond converts single-chain tPA (sc-tPA) into the canonical 2-chain form (tc-tPA) [2], [3]. Thus, tPA is secreted as sc-tPA and can be processed into tc-tPA by plasmin or kallikreins. Unlike many other serine proteases, tPA is proteolytically active as both sc-tPA and tc-tPA, although tc-tPA is approximately 3- to 5-fold more active than sc-tPA [4], [5]. The thrombolytic drug alteplase contains both forms of tPA [6].

tPA also has functions beyond its thrombolytic activity, including roles in cell survival, vessel-wall homeostasis, and inflammatory processes [7], [8]. Although sc-tPA and tc-tPA have broadly similar thrombolytic properties in the presence of fibrin [9], some nonfibrinolytic effects of tPA appear to depend on its chain form [10], [11].

In ischemic stroke, intravenous administration of tPA within 4.5 hours of symptom onset improves clinical outcomes and remains a standard acute reperfusion treatment. This benefit is primarily attributed to early recanalization. In a previous clinical study, although the sc-tPA/(sc-tPA + tc-tPA) ratio was not associated with overall outcome after cerebral ischemia, early seizures were more frequent in patients who received preparations with a sc-tPA/(sc-tPA + tc-tPA) ratio greater than 80.5% [6]. Tenecteplase (TNK), another clinically used thrombolytic, is also administered as a mixture of single-chain TNK (sc-TNK) and the 2-chain form (tc-TNK) [12].

In this *in vitro* study, including experiments with human plasma and whole blood, we investigated how the tPA chain form affects fibrinolytic function. Guided by phylogenetic analysis, we generated a set of tPA mutants targeting residues around the canonical cleavage site. Our data support distinct properties of sc-tPA and tc-tPA and reveal an alternative 2-chain (tc) form, tc(K277)-tPA, generated by cleavage between K277 and G278 when R275 is mutated. These findings provide mechanistic insight into tPA processing and identify a basis for further preclinical evaluation of tPA-related variants.

## 2 Methods

### 2.1 Multiple sequence alignment

RefSeq proteins (1 sequence per gene) for the genes PLAT, PLAU, HGFA, and HABP2 were obtained from the National Center for Biotechnology Information Orthologs database (January 23, 2025). Multiple sequence alignments (MSAs) were performed with Clustal Omega (default settings) [13] in Jalview software (version 2.11.4.1) [14]. Only the amino acid sequences surrounding the cleavage site of each protein were retained (corresponding to Q273-G279 in human tPA). Results were presented as sequence logos generated with WebLogo (version 2.8.2) [15] without small-sample correction. A phylogenetic tree was constructed according to previous literature [16] and the National Center for Biotechnology Information database. Animal drawings were sourced from BioRender.com. Amino acid numbering of human tPA [17], [18] served as the reference for all tPA-related proteins.

### 2.2 Electrophoresis and Coomassie staining

To quantify purified protein and the forms (sc-tPA/tc-tPA) of purified tPA variants, electrophoresis was performed. Proteins were resolved by sodium dodecyl sulfate–polyacrylamide gel electrophoresis on 10% or 12% polyacrylamide gels (4568033/4568043/4568046, Bio-Rad). Proteins in gels were stained with Coomassie blue and imaged with the ChemiDoc MP Imaging System (Bio-Rad). Purified protein quantification was estimated by densitometric analysis using ImageLab software (Bio-Rad).

### 2.3 Western blot

Resistance to plasmin cleavage and plasma-mediated cleavage was assessed by Western blot analysis. Primary antibodies against tPA (ab62763, Abcam) and against the tPA heavy chain (IMSAHUTPAHC2A153C1MG, Innovative Research) were usedSupplementary Figure S6. Details are provided in the Supplementary Methods.

### 2.4 Enzymatic activity

#### 2.4.1 Amidolytic activity

Amidolytic activity of purified tPA variants was assessed using a fluorogenic substrate (Pefafluor tPA (PF tPA, CH_3_SO_2_-D-Phe-Gly-Arg-AMC⋅AcOH, 091-06, Interchim Bioscience)). Details are provided in the Supplementary Methods.

#### 2.4.2 Plasminogen-fibrin overlay zymography

Indirect amidolytic activity was measured by zymography in the presence of plasminogen and fibrin. Plasminogen-fibrin overlay zymography was performed using a modified version of previously described methods [19], [20]. Details are provided in the Supplementary Methods.

### 2.5 Clot lysis assay

#### 2.5.1 Plasma-based clot formation and lysis assay

Fibrin-rich clot lysis capacity was evaluated using a plasma-based clot formation and lysis assay. Citrated plasma from 8 healthy volunteer donors (Établissement Français du Sang, convention 2024/174) was pooled and centrifuged at 1500 × *g* for 15 minutes at room temperature to obtain platelet-poor plasma (PPP). PPP was then stored in aliquots at −80 °C. Shortly before the assay, PPP was briefly thawed at 37 °C and then placed on ice. Purified tPA variants were diluted to a final volume of 20 μL in HEPES (4-(2-hydroxyethyl)-1-piperazineethanesulfonic acid) buffer (10 mM HEPES, 150 mM NaCl [S3014, Sigma-Aldrich], 0.001% v/v Tween 20 [P1379, Sigma-Aldrich], pH 7.4) and then mixed with PPP (25 μL) at different concentrations in a 96-well clear flat-bottom plate (655101, Greiner Bio-One). Next, 5 μL of calcium solution (250 mM CaCl_2_ [C1016, Sigma-Aldrich]) was added to trigger fibrin-rich clot formation. Plates were sealed with a clear film (E2796-9795, Starlab Group) prior to acquisition. Absorbance was measured at 405 nm every 30 seconds for 18 hours at 37 °C in a plate reader (Spark Multimode Microplate Reader, Tecan). All experiments were conducted in triplicate, and only the means were retained for analysis. All tested tPAs were run on the same plate in different positions to minimize positional effects. Each plate included positive controls (PPP + HEPES buffer + calcium solution) and negative controls (PPP + HEPES buffer) to validate clot formation. Turbidimetric curves recorded at 405 nm were processed using a spreadsheet macro developed in our laboratory by Dr Maxime Gauberti to automate data extraction. The 50% clot lysis time (hereafter referred to as clot lysis time) was calculated as the time required for a 50% reduction in turbidity, from maximum absorbance to the postlysis baseline, as previously described [21], [22].

#### 2.5.2 Rotational thromboelastometry

Blood clot lysis efficiency was assessed using rotational thromboelastometry (ROTEM). Citrated fresh whole blood, collected within 1 hour of sampling from 32 healthy volunteer donors (Établissement Français du Sang), was analyzed on the ROTEM delta system (Werfen) according to the manufacturer’s instructions. One tPA variant (0-10 nM final) was tested per blood donor. Blood (final volume 350 μL) was mixed with tPA diluted in a saline solution (0.9% w/v NaCl). The mixture (300 μL) was then injected into the Cup & Pin Pro (200011, Werfen) with 20 μL of r ex-tem reagent (containing recombinant tissue factor, phospholipids, heparin inhibitor, preservatives, and buffer; 503-05, Werfen) and 20 μL of star-tem reagent (containing CaCl_2_, preservatives, and buffer; 503-10, Werfen). The firmness of the blood was measured at 37 °C for 2 hours. Acquisitions were exported and analyzed in Microsoft Excel (Microsoft Corporation). ROTEM parameters provided automatically were verified visually. Fibrinolysis speed and corrected fibrinolysis speed were calculated as previously described [23].

### 2.6 Statistical analysis

Normal distribution was assessed using the Shapiro–Wilk test in GraphPad Prism. Homogeneity of variances was evaluated using the Brown–Forsythe test (α = .05) in Microsoft Excel (Microsoft Corporation). For multiple group comparisons of nonnormally distributed data, we performed a Kruskal–Wallis test followed by Dunn’s multiple comparison test (comparing either to a control condition or across all groups) with an α level of .05 in GraphPad Prism (version 10.3.0). Data are expressed as mean ± SD, except for curves, which are shown as mean or individual curves.

## 3 Results

### 3.1 Phylogenetic analysis of the tPA cleavage site

To explore the evolutionary significance of the tPA cleavage region, focusing on the previously described R275-I276 cleavage site [3], we constructed a phylogenetic tree based on MSA (Figure 1A). The MSA showed that R275 is highly conserved among *Gnathostomata* (jawed vertebrates; 95.8%), including early-diverging groups such as *Chondrichthyes* (cartilaginous fishes; Figure 1A, B). Among 645 species, 20 exhibited a substitution at R275 (Figure 1C), potentially affecting this cleavage site. A second potential cleavage site, 2 amino acids downstream of R275, was previously reported by Higgins et al. [24] between K277 and G278. Although K277 is conserved in 60.5% of all species, species with a substitution at R275 generally retained K277. The only exceptions were the delta smelt, *Hypomesus transpacificus* (*Actinopterygii*), and the common vampire bat, *Desmodus rotundus* (*Mammalia*); the latter is the only tPA reported to resist plasmin cleavage [25]. Notably, in *Serpentes* (snakes), all tPA molecules possessed only the K277-G278 cleavage site (Figure 1A, C). K277 was also more conserved among *Tetrapoda* (86.7%; Figure 1A). Together, these observations are consistent with the presence of a second potential cleavage site between K277 and G278. Close paralogs (urokinase-type plasminogen activator, HGFA, and HABP2) consistently exhibited a conserved arginine or lysine at the P1 site (>97%) but lack these residues at the P2' site (Supplementary Figure S1A). The only exceptions are urokinase-type plasminogen activator from domestic sheep (*Ovis aries*) and HGFA from the microbat (*Myotis davidii*), which lack arginine or lysine at P1 (Supplementary Figure S1B). Finally, predictive 3-dimensional modeling of human tPA indicated that K277 is near R275 and exposed on the protein surface (Figure 1B).

**Figure 1:**
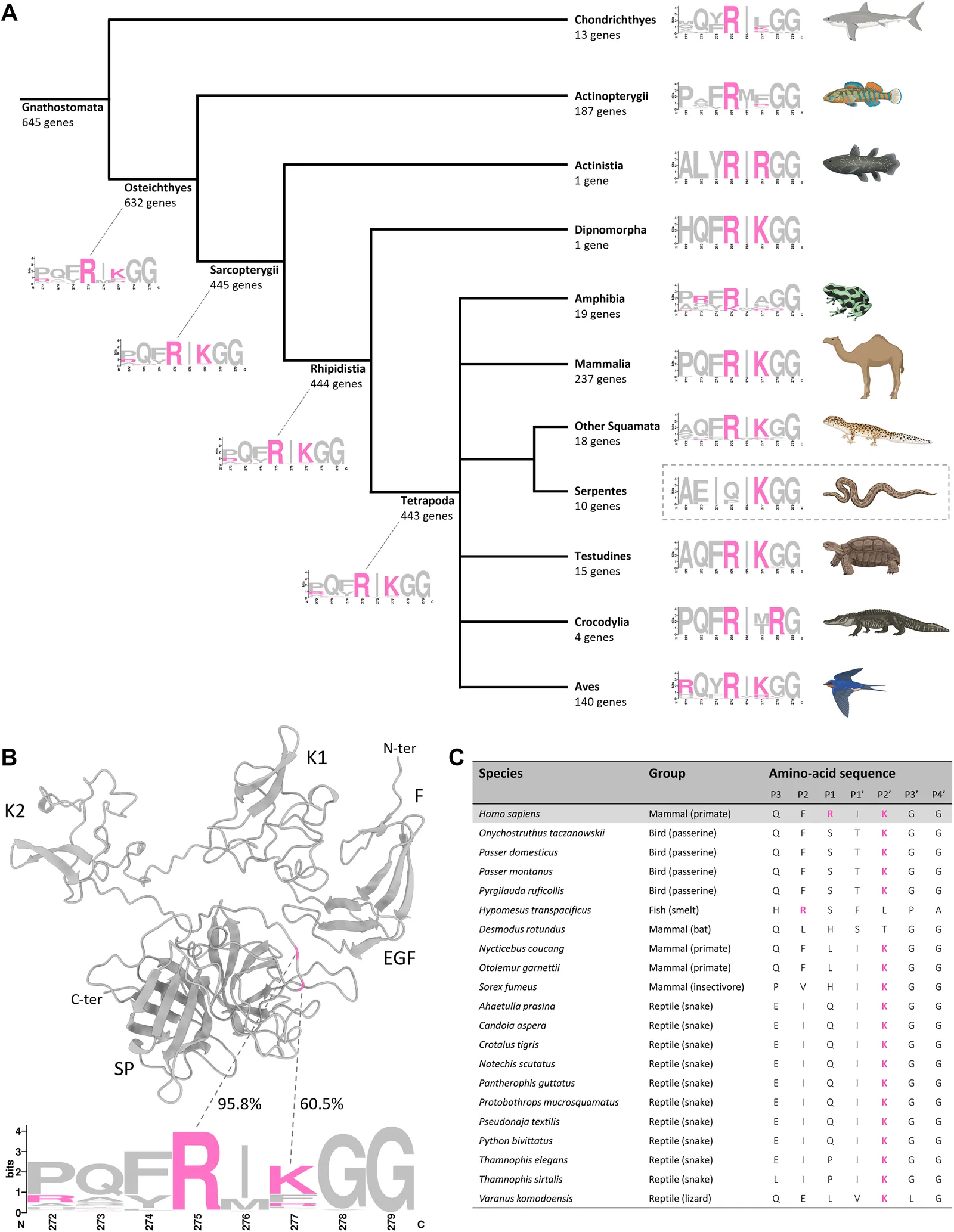
Snake tissue plasminogen activators (tPAs) have only the second tPA cleavage site. (A) Phylogeny of *Gnathostomata* with the consensus sequence surrounding the tPA cleavage site. The number of PLAT genes used is indicated under the group name. Animal pictures are from BioRender.com. The *Serpentes* group is highlighted with a gray dotted rectangle. R and K amino acids are shown in pink. (B) Upper part: 3-dimensional predicted structure of human tPA (P00750.1) using the Colab version of AlphaFold 2.1, represented as a cartoon backbone. C-ter, C-terminus; EGF, epidermal growth factor domain; F, finger domain; K, kringle domain; N-ter, N-terminus; SP, serine protease domain. R275 and K277 are shown in pink. Lower part: WebLogo generated from a multiple sequence alignment of all tPAs from *Gnathostomata* between X272 and X279. R and K amino acids are shown in pink, with associated homology percentages. (C) Sequence alignment surrounding the cleavage site of tPA from species with R275 mutated. Human tPA is highlighted in light gray. R and K amino acids are shown in pink. R275 of human tPA is referred to as the P1 amino acid.

### 3.2 An unexpected alternative tPA cleavage site

Based on these findings, we generated human tPA mutants carrying either a single substitution at R275 (R275G or R275S) or a double substitution at R275 and K277 (R275G-K277S and R275S-K277S) to investigate whether an alternative tc-tPA form could be produced (Figure 2A). All mutant human tPAs were expressed as sc-tPA (100%; Figure 2B), whereas wild-type (WT) human tPA was predominantly expressed as sc-tPA (72% ± 4%; Figure 2B). The concentration of each purified tPA variant was quantified by densitometric analysis of Coomassie blue-stained sodium dodecyl sulfate–polyacrylamide gel electrophoresis gels, using clinical-grade alteplase as an internal standard over a linear calibration range (Supplementary Figure S2A, B). Resistance to plasmin-mediated cleavage was assessed by incubating tPA variants with human plasmin at varying concentrations, followed by Western blot analysis and quantification (Figure 2C). WT-tPA was efficiently converted to tc-tPA, even at low plasmin concentrations (22% ± 9% tc-tPA without plasmin vs 60% ± 16% tc-tPA with 0.04 μg of plasmin). Single mutants R275G and R275S showed bands with an apparent molecular weight similar to that of the tc-tPA bands of WT-tPA (approximately 30-35 kDa). Quantification showed reduced cleavage by plasmin compared with WT-tPA (86% ± 11% tc-tPA for WT vs 30% ± 10% for R275G and 21% ± 6% for R275S with 0.2 μg of plasmin). Double mutants were resistant to cleavage, with minimal tc-tPA formation (7% ± 10% for R275G-K277S and 5% ± 5% for R275S-K277S with 0.2 μg of plasmin). These results support the presence of a second cleavage site near the canonical R275-I276 site and implicate K277 in this processing event. To map the cleavage site more directly, we performed Edman degradation on selected tPA mutants after incubation with plasmin (Figure 2D). We selected tPA variants with the greatest apparent resistance to plasmin cleavage to improve detection of specific cleavage events. As expected, the N-terminal sequences of the A-chain in WT, R275S, and R275S-K277S tPAs began with Gly-Ala-Arg-His-, with Gly corresponding to G(-3), and His is the first residue of the 6×His tag used for purification (see Supplementary Methods). The N-terminal sequence of the B-chain in WT-tPA began with Ile-Lys-Gly-Gly-, as previously reported [3], whereas the B-chain of R275S began with Gly-Gly-Leu-Phe-, with the first glycine corresponding to G278. No B-chain was detected for R275S-K277S. Together, these findings confirm the primary cleavage site between R275 and I276 and support an alternative cleavage site between K277 and G278.

**Figure 2:**
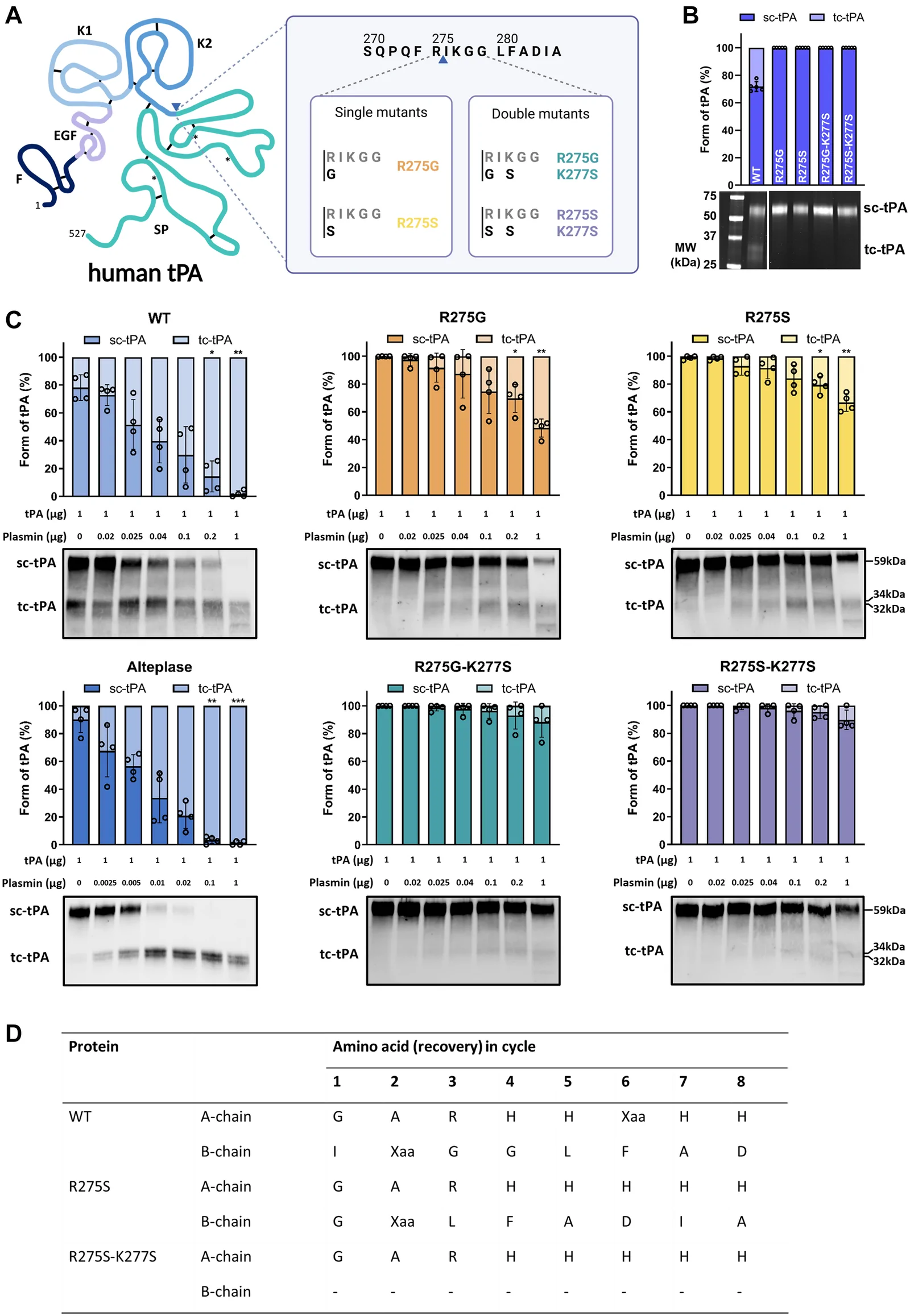
Plasmin cleaves tissue plasminogen activator (tPA) after R275 and K277. (A) Left part: schematic representation of mature human tPA. ▲, cleavage site; ∗, active site residues; black lines, disulfide bonds; EGF, epidermal growth factor domain; F, finger domain; K, kringle domain; SP, serine protease domain; based on Ny et al. [18]. Right part: overview of generated tPA mutants surrounding the cleavage site. Created with BioRender.com. (B) Lower part: representative reducing sodium dodecyl sulfate–polyacrylamide gel electrophoresis followed by Coomassie blue staining of tPA variants. Molecular weight (MW) markers are indicated on the left in kilodaltons (kDa). Upper part: proportion of single-chain tPA (sc-tPA) and 2-chain tPA (tc-tPA) variants. Data represent the mean ± SD of 5 independent experiments. (C) Western blot of tPA variants and alteplase (1538 nM final concentration; 1 μg loaded per lane) after 1 hour of incubation at 37 °C with increasing quantities of plasmin (0, 3, 6, 12, 24, 30, 48, 120, 241, and 1205 nM, corresponding to 0, 0.0025, 0.005, 0.01, 0.02, 0.025, 0.04, 0.1, 0.2, and 1 μg loaded per lane, respectively). Primary antibodies against tPA (ab62763, Abcam) were used. Data represent the mean ± SD of 4 independent experiments. Comparisons were performed using the Kruskal–Wallis test followed by Dunn's post hoc multiple comparison test, with the tPA without plasmin condition as the reference group. ∗*P* < .05, ∗∗*P* < .01, ∗∗∗*P* < .001. (D) N-terminal sequences of the A- and B-chains of tPA variants after 1 hour of incubation at 37 °C with 5 times less plasmin, as revealed by Edman sequencing. -, no signal detected; Xaa, unidentified amino acid residue; WT, wild-type.

### 3.3 The tPA form governs enzymatic activity

It has previously been shown that sc-tPA and tc-tPA exhibit distinct enzymatic properties [9], [26]. However, many assays use plasminogen or plasmin, both of which can cleave tPA. We therefore generated noncleavable tPA variants with an R275 mutation to more precisely characterize enzymatic activity and explore the fibrinolytic properties of these mutants [4]. To generate tc-tPA from WT-tPA, we incubated WT-tPA with plasmin-coated beads (Supplementary Figure S3). From this point forward, WT-tPA containing 72% ± 4% sc-tPA is referred to as sc-WT, whereas WT-tPA containing 91% ± 2% tc-tPA is designated tc-WT (Supplementary Figure S3). Amidolytic activities were assessed using a tPA fluorogenic tripeptide substrate (Figure 3 and Michaelis–Menten curves in Supplementary Figure S4). In the absence of coactivators, all mutants exhibited similar catalytic efficiency (specificity constant), except R275G-K277S, which showed reduced activity (*P* < .001 vs sc-WT; Figure 3A). Sc-WT demonstrated approximately 3-fold higher activity than the mutants but approximately 4-fold lower activity than tc-WT (Figure 3A). The addition of human plasminogen activator inhibitor-1 (PAI-1) resulted in approximately a 2-fold reduction in the activity of sc-WT and mutant tPAs, with no detectable effect on tc-WT activity (Figure 3B). To assess enzymatic activity in the presence of coactivators, we performed plasminogen-fibrin overlay zymography (Figure 3C). Quantification of band intensity at 60 minutes showed similar activity levels for sc-WT, tc-WT, R275G, and R275S, whereas double mutants showed approximately 2-fold lower activity throughout the incubation period (Figure 3D). Lysis velocity, derived from band area over time, further supported these findings (Figure 3E). Sc-WT (0.40 ± 0.14 mm^2^/min) and tc-WT-tPA (0.40 ± 0.10 mm^2^/min) had similar average lysis velocities. Single mutants showed comparable average lysis velocities (R275G: 0.29 ± 0.05 mm^2^/min; R275S: 0.36 ± 0.10 mm^2^/min), whereas double mutants showed approximately 2-fold lower lysis velocities (R275G-K277S: 0.19 ± 0.06 mm^2^/min; R275S-K277S: 0.20 ± 0.04 mm^2^/min).

**Figure 3:**
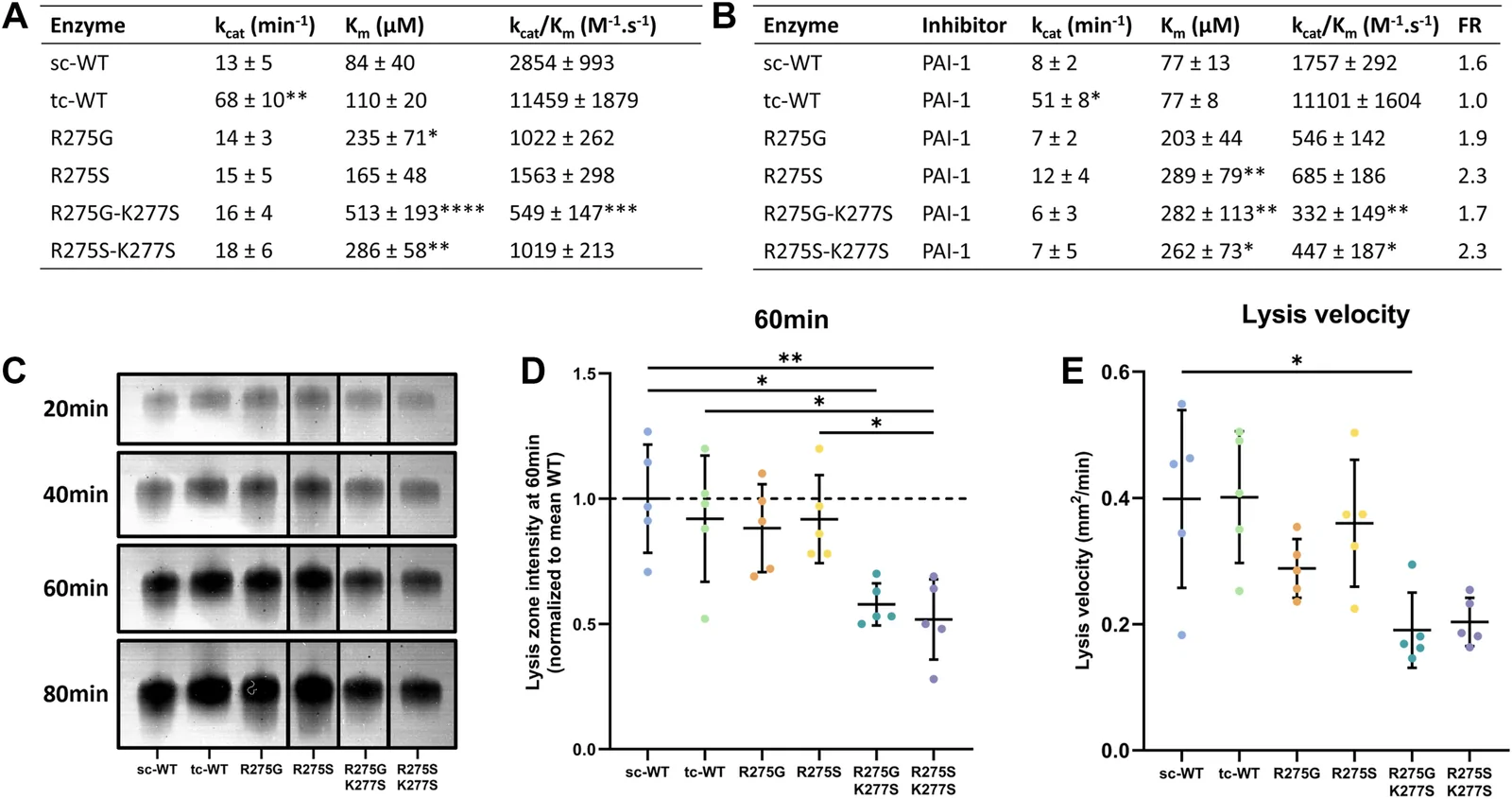
Enzymatic profiles of the different forms of tissue plasminogen activator (tPA). (A) Kinetic constants from the Michaelis–Menten curves (Supplementary Figure S3) for the hydrolysis of Pefafluor-tPA (PF-tPA) at increasing concentrations of tPA variants (2 μg/mL [≈30.8 nM]) at 37 °C. Data represent the mean ± SD of 6 independent experiments, each performed in duplicate. Comparisons were performed using the Kruskal–Wallis test followed by Dunn's post hoc multiple comparison test, with the single-chain wild type tPA (sc-WT) condition as the reference group. ∗*P* < .05, ∗∗*P* < .01, ∗∗∗*P* < .001, ∗∗∗∗*P* < .0001. (B) Kinetic constants from the Michaelis–Menten curves (Supplementary Figure S3) for hydrolysis of PF-tPA at increasing concentrations of tPA variants (2 μg/mL [≈30.8 nM]) and plasminogen activator inhibitor-1 (PAI-1; 2 μg/mL [≈41.7 nM]) at 37 °C. Prior to the addition of PF-tPA, tPA variants and plasminogen activator inhibitor-1 were incubated for 10 minutes at 37 °C. Data represent the mean ± SD of 6 independent experiments, each performed in duplicate. Comparisons were performed using the Kruskal–Wallis test followed by Dunn's post hoc multiple comparison test, with the sc-WT condition as the reference group. ∗*P* < .05, ∗∗*P* < .01. FR, fold reduction ratio. (C) Representative plasminogen-fibrin overlay zymography of tPA variants (0.2 μg/lane), acquired every 20 minutes over 80 minutes at 37 °C. Acquisitions were acquired identically and are shown with the same brightness and contrast parameters. (D) Lysis zone intensity of tPA variants at 60 minutes of incubation at 37 °C normalized to the mean lysis zone intensity of sc-WT from plasminogen-fibrin overlay zymography. Data represent the mean ± SD of 5 independent experiments. Comparisons were performed using the Kruskal–Wallis test followed by Dunn's post hoc multiple comparison test. ∗*P* < .05, ∗∗*P* < .01. (E) Lysis velocity of tPA variants from plasminogen-fibrin overlay zymography was determined by measuring the area of the lysis zones at 37 °C as a function of incubation time. Data represent the mean ± SD of 5 independent experiments. Comparisons were performed using the Kruskal–Wallis test followed by Dunn's post hoc multiple comparison test. ∗*P* < .05. WT, wild-type. k_cat_, catalytic constant; k_cat_/K_m_, specificity constant; K_m_, Michaelis constant; tc-WT, 2-chain wild type tPA.

### 3.4 Implication of the second cleavage site in clot lysis

To assess the clot lysis capacity of our tPA variant set, we performed a plasma-based lysis assay using pooled citrated PPP to generate fibrin-rich clots sensitive to tPA-mediated lysis (Figure 4). tPA variants were added to PPP at concentrations ranging from 0.75 to 1.50 nM prior to coagulation initiation with calcium ions to measure clot absorbance (Figure 4A). Single mutants (R275G and R275S) exhibited significantly faster lysis kinetics, dissolving 50% of the clot in approximately 1.9- and 2.4-fold less time (for R275G and R275S, respectively) than sc-WT, averaged across all concentrations (Figure 4B). In contrast, double mutants showed lysis kinetics almost similar to sc-WT (Figure 4B).

**Figure 4:**
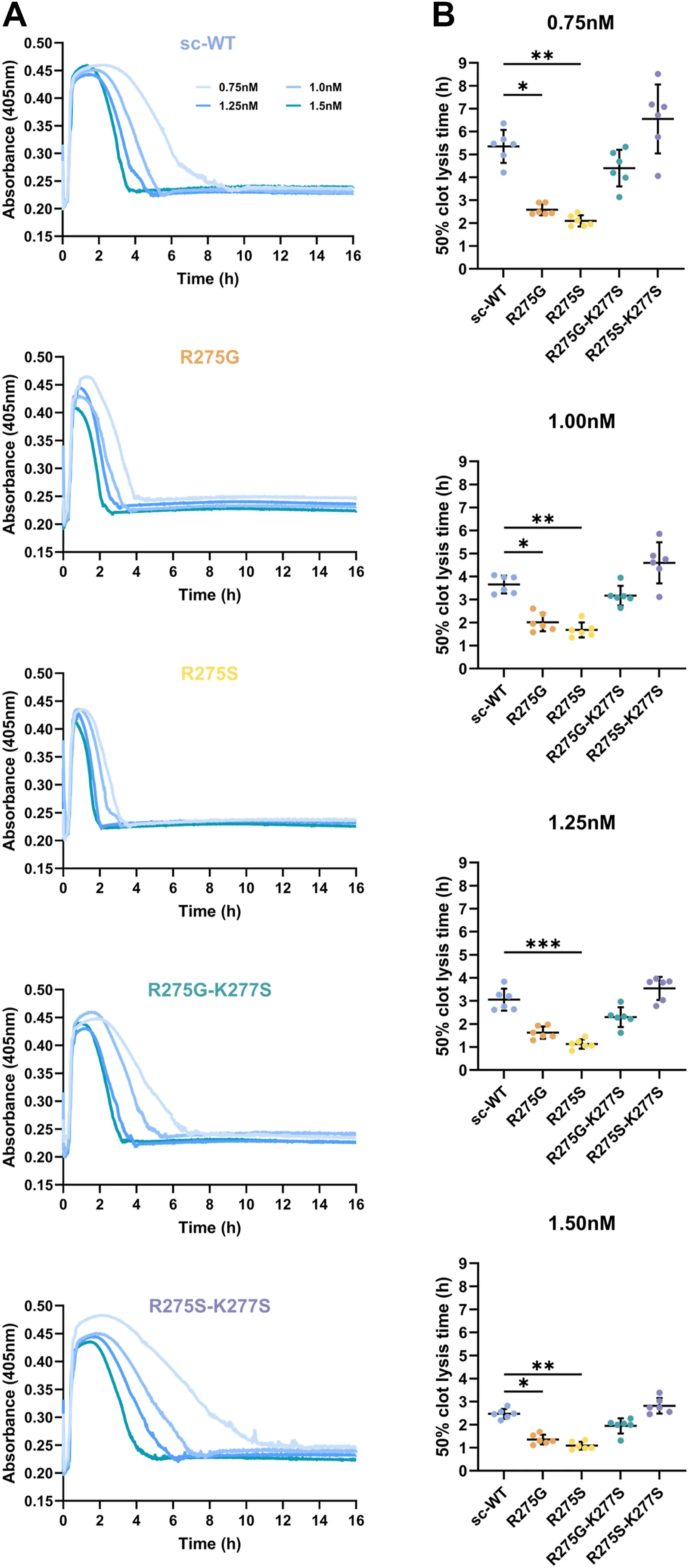
Single mutants of tissue plasminogen activator (tPA; single-chain [sc]-R275S or sc-R275G) show higher efficacy in dissolving fibrin-rich clots. (A) Absorbance curves from the clot lysis assay using platelet-poor plasma and tPA variants at increasing concentrations at 37 °C. Data represent the mean curves of 6 independent experiments, each performed in triplicate. (B) 50% clot lysis time, measured by the clot lysis assay using platelet-poor plasma and tPA variants at concentrations of 0.75 to 1.50 nM. Data represent the mean ± SD of 6 independent experiments, each performed in triplicate. Comparisons were performed using the Kruskal–Wallis test followed by Dunn’s post hoc multiple comparison test, with the sc-wild-type (WT) condition as the reference group. ∗*P* < .05, ∗∗*P* < .01, ∗∗∗*P* < .001.

To clearly distinguish the initial molecular state of the input material across subsequent experiments, we hereafter designated the purified, uncleaved single mutants (containing 100% sc-tPA at baseline) as sc-R275G and sc-R275S.

We then used ROTEM to assess how sc-tPA variants affect clot lysis when present during clot formation (Figure 5). ROTEM profiles at the highest tPA concentration (10 nM) are shown in Figure 5A, with additional lower concentration profiles in Supplementary Figure S5A. Detailed analysis of ROTEM parameters is provided in Figure 5B and Supplementary Figure S5B. For lysis time, sc-R275S showed the strongest effect among the tested single-chain (sc) variants, lysing 90% of the clot (lysis time parameter) in approximately half the time required by sc-WT-tPA at 10 nM (Figure 5B). The sc-R275S mutant also showed the fastest fibrinolysis rates and corrected lysis speeds, approximately 2- to 3-fold faster than sc-WT under these conditions. In contrast, double mutants consistently showed longer lysis curves, indicating reduced fibrinolytic capacity compared with the other variants tested.

**Figure 5:**
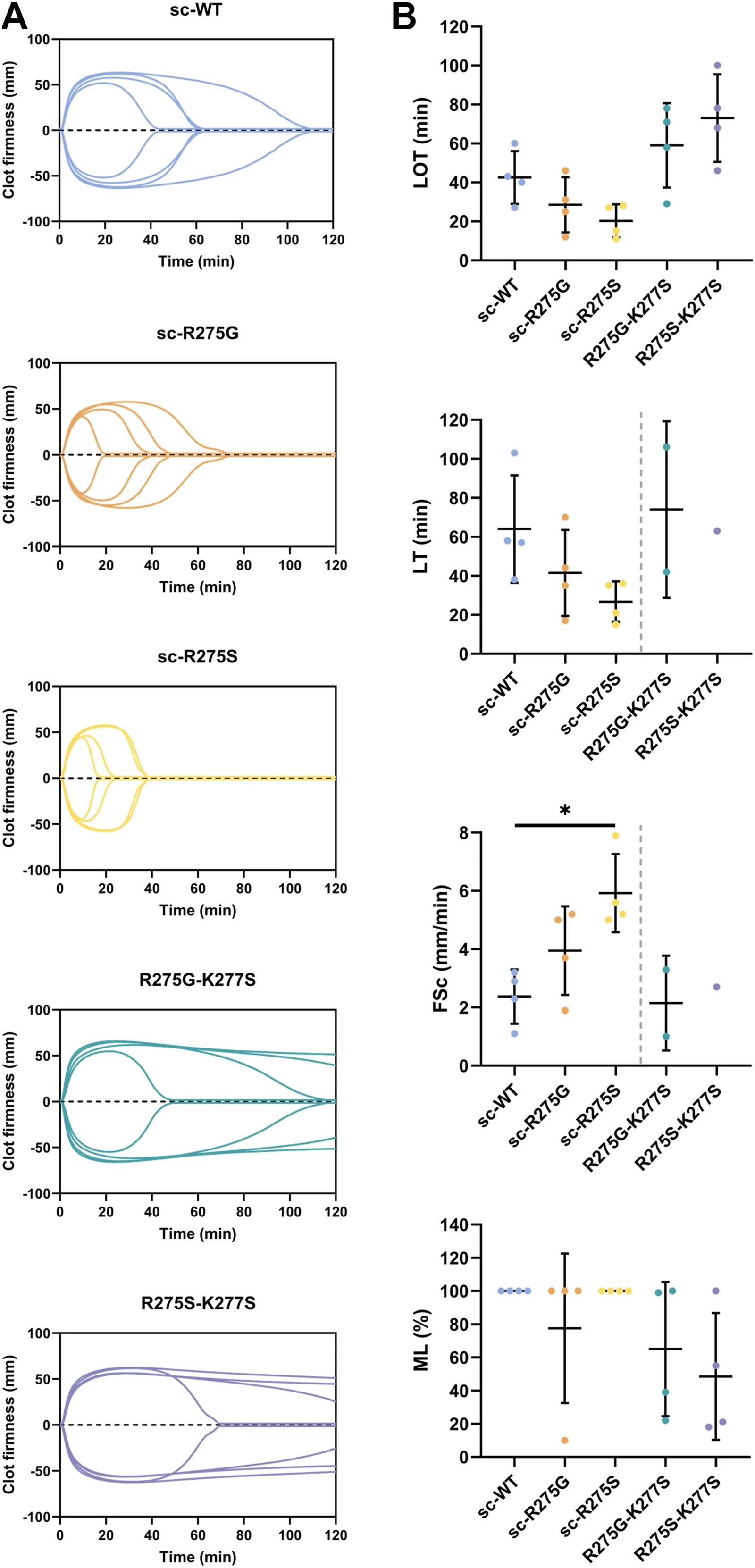
Single mutants of tissue plasminogen activator (tPA; single-chain [sc]-R275S or sc-R275G) show a greater capacity to dissolve whole blood clots. (A) Rotational thromboelastometry (ROTEM) profiles of clot firmness in whole blood mixed with r ex-tem/star-tem, and sc-tPA variants at 10 nM. Each curve represents an individual blood donor. Experiments were performed 4 times for each tPA variant. (B) ROTEM parameters obtained from ROTEM curves in (A) with sc-tPA variants at 10 nM. Data represent the mean ± SD of 4 independent experiments for each tPA variant. Comparisons were performed using the Kruskal–Wallis test followed by Dunn’s post hoc multiple comparison test, with the sc-wild-type (WT) condition as the reference group, except for conditions with *n* < 3 (indicated by vertical gray dashes). ∗*P* < .05. FSc, corrected fibrinolysis speed; LOT, lysis onset time; LT, lysis time; ML, maximum lysis.

### 3.5 Cleavage in plasma

We next incubated alteplase, sc-WT, sc-R275S, and R275S-K277S with human plasma (Supplementary Figure S6A) to determine whether conversion of sc-tPA to tc-tPA via cleavage after R275 or K277 could occur under plasma conditions. The addition of tPA variants to plasma produced a distinct band at approximately 35 kDa, corresponding to the tPA heavy chain of alteplase, sc-WT, and sc-R275S. For these 3 variants, band intensity increased over time, most notably between 3 and 15 minutes. In contrast, no band at approximately 35 kDa was detected for R275S-K277S, consistent with the absence of detectable cleavage under these assay conditions. The specificity of the antibody used to detect the tPA heavy chain was verified in Supplementary Figure S6B. Overall, these data indicate that tPA can undergo proteolytic cleavage at both R275 and K277 in human plasma.

### 3.6 Functional assessment of Lys277-cleaved tc-tPA preparations

We continued our investigations using the tc forms of WT-tPA, R275G, and R275S. Prior to these assays, the sc variants sc-R275G and sc-R275S were incubated with plasmin-coated beads to induce conversion to their tc forms. This procedure resulted in a 50:50 sc/tc mixture for R275G and a 79:21 sc/tc mixture for R275S (Supplementary Figure S3). These partially processed preparations were designated sc/tc-R275G and sc/tc-R275S, respectively.

The fibrinolytic capacity of tc-WT and the sc/tc single-mutant tPA preparations was quantified. At 10 nM, sc/tc-R275S produced the shortest ROTEM profiles compared with those of tc-WT and sc/tc-R275G (Figure 6A). Accordingly, sc/tc-R275S showed enhanced fibrinolytic efficacy, with a statistically significant improvement in corrected fibrinolysis speed over tc-WT (2.8-fold higher than tc-WT and 1.6-fold higher than sc/tc-R275G; Figure 6B). Consistent with this effect, numerical trends toward faster lysis were observed for other ROTEM metrics, although these differences did not reach statistical significance. The time to initiate lysis (15% reduction in clot firmness) and the time to achieve a 90% reduction in clot firmness were numerically lowest for sc/tc-R275S, with lysis onset time approximately half that observed for tc-WT. Results obtained at 4.6 and 6.9 nM tPA showed similar trends (Supplementary Figure S7A, B).

**Figure 6:**
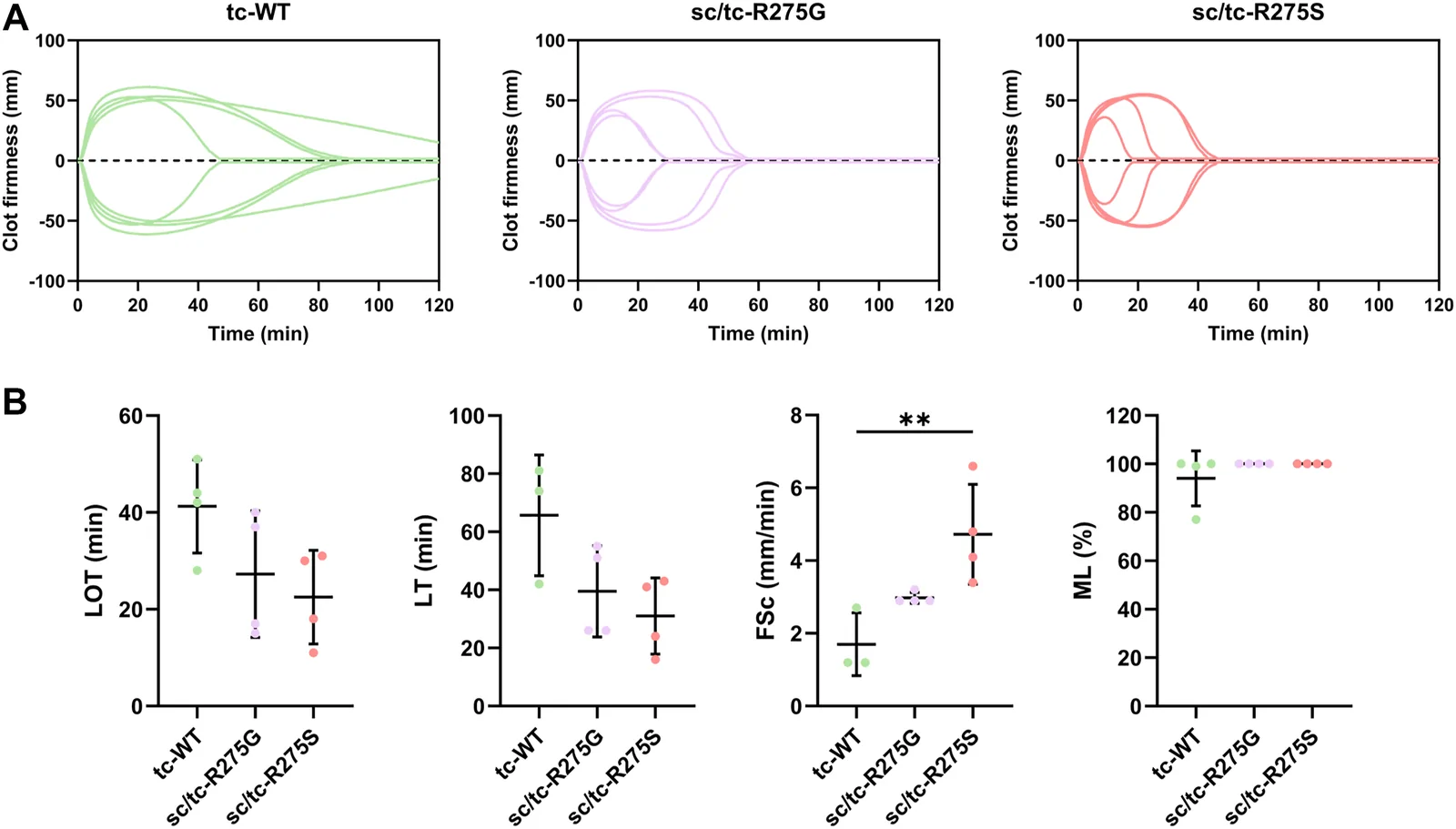
Fibrinolytic activity of 2-chain tissue plasminogen activator (tc-tPA) variants. (A) Rotational thromboelastometry (ROTEM) profiles of whole blood clot firmness with r ex-tem/star-tem and tc-tPA variants at 10 nM. Each curve represents an individual blood donor. Experiments were performed 4 times for each tissue plasminogen activator variant. (B) ROTEM parameters derived from the ROTEM curves in (A) for tc-tPA variants at 10 nM. Data represent the mean ± SD of 4 independent experiments per tissue plasminogen activator variant. Comparisons were performed using the Kruskal–Wallis test followed by Dunn’s post hoc multiple comparison test, with 2-chain wild type tPA (tc-WT) as the reference group. ∗∗*P* < .01. FSc, corrected fibrinolysis speed; LOT, lysis onset time; LT, lysis time; ML, maximum lysis; sc, single-chain; tc, 2-chain.

## 4 Discussion

tPA exists in 2 principal molecular forms: sc-tPA and tc-tPA. Although both forms promote fibrinolysis, they have distinct biochemical properties that may influence physiological and therapeutic responses. Conversion of sc-tPA to tc-tPA is primarily mediated by plasmin, although other proteases, including kallikrein and follicular serine proteases, can participate under specific physiological conditions [2], [27], [28]. Although sc-tPA has intrinsic catalytic activity, it behaves like a zymogen and requires allosteric activation, typically through fibrin binding, to achieve full activity [4], [5]. Domain-specific interactions and posttranslational modifications, particularly glycosylation, further modulate chain-specific functions [29], [30]. The finger and kringle 2 domains contribute to fibrin affinity [31] and to spatial positioning within the fibrin matrix, thereby facilitating plasminogen activation [32], [33]. Our findings indicate that residues R275 and K277 contribute to tPA processing and fibrinolytic activity in the experimental systems studied here.

Phylogenetic analysis of 645 species revealed that 20 had an R275 substitution, potentially disrupting the canonical cleavage site. Across all snake genomes studied, tPA variants contain only K277, and K277 conservation was higher across *Tetrapoda* (86.7%). Moreover, Higgins et al. [24] previously reported a cleavage site between K277 and G278, supporting our *in vitro* evidence for a secondary cleavage site. Although K277 is conserved in only 60.5% of species, species lacking R275 generally retain K277, except *H transpacificus* and *D rotundus*; the latter is the only tPA known to resist plasmin cleavage [34], [35]. UniProt lists 4 human variants at R275 (R275C, R275H, R275L, and R275P), with no likely pathogenic or pathogenic annotation reported to date. Together, these observations suggest that K277 can serve as an alternative cleavage site when R275 is absent and support further evaluation of these tPA forms in fibrinolysis.

A key functional distinction between sc-tPA and tc(R275)-tPA is their fibrin dependence: sc-tPA requires fibrin binding for full activation, whereas tc(R275)-tPA shows greater fibrin-independent activity. In fibrin-rich environments, sc-tPA can achieve activity comparable to that of tc(R275)-tPA through allosteric enhancement [26]. Conversely, the fibrin-independent activity of tc(R275)-tPA may contribute to rapid activity in thrombolytic settings, although the clinical relevance of this distinction depends on context and requires direct evaluation.

Based on these insights, we engineered human tPA mutants harboring single (R275G or R275S) or double (R275G-K277S or R275S-K277S) substitutions. Plasmin cleavage assays confirmed that WT-tPA is cleaved at the R275-I276 site, as previously reported [2], [3]. Mutation at R275 led to the generation of an alternative tc form, referred to here as tc(K277)-tPA, which is distinct from tc(R275)-tPA and associated with enhanced fibrinolytic activity in the assays performed. Preparations enriched in tc(K277)-tPA (sc/tc-R275G/S) consistently showed higher fibrinolytic activity than tc(R275)-tPA (tc-WT). However, because these mutant preparations are mixtures of sc and Lys277-cleaved isoforms rather than homogeneous tc entities, their overall fibrinolytic performance reflects the combined activity of both species under fibrin-stimulated conditions.

In clot lysis assays, single mutants showed variable effects on fibrinolytic activity, consistent with previous observations [36]. These differences may partly arise from the distinct physicochemical properties of the substituted amino acids (Gly vs Ser) [37]. Structural considerations may also contribute. Indeed, the N-terminal ammonium group of Ile276 is known to play a key conformational role by stabilizing the active tc form through formation of a salt bridge with Asp477, which is functionally linked to the catalytic site [38]. Therefore, substitution at position 275 could indirectly affect the positioning or stability of I276, thereby modulating the final enzymatic activity of mutant proteins. It is important to note that even a single mutation in tPA may induce multiple structural and functional changes that affect its 3-dimensional conformation and intermolecular interactions [39].

Our results also support a previous study showing that in buffer, sc-tPA is inhibited by PAI-1 more rapidly than tc-tPA [26]. The same study [26] further demonstrated that, in the presence of fibrin, both sc-tPA and tc-tPA are only minimally inhibited by PAI-1, and to a similar extent. Building on these observations, it would be informative to evaluate PAI-1–mediated inhibition of sc-tPA, tc(R275)-tPA, and tc(K277)-tPA under fibrin-rich conditions. The absence of a K277 single mutant may be a limitation of the present study. However, because K277 is located downstream of the canonical R275 cleavage site, we would not expect mutation of K277 alone to substantially alter tPA processing or function under physiological conditions, where cleavage predominantly occurs at R275. Our data rather support the concept that K277 acts as a latent or backup cleavage site that becomes functionally relevant only when the canonical R275 cleavage site is disrupted.

Consistent with previous findings [24] and our data, sc-WT-tPA is cleaved more rapidly by plasmin than the sc-R275G/S variants. Several factors may account for this difference, including sequence recognition at the cleavage site [40], the 3-dimensional accessibility of the 2 cleavage sites, and/or the presence of cofactors such as fibrin(ogen), which interacts with both tPA and plasmin [41].

Remarkably, our findings demonstrate that sc-WT-tPA, and to a lesser extent sc-R275S, undergoes sc-to-tc conversion directly in plasma in the absence of exogenous clot activators. This conversion in resting plasma aligns with recent findings [42], suggesting that fibrinogen may serve as a cofactor for tPA-dependent plasminogen activation and subsequently mediate sc-tPA cleavage.

Further studies quantifying the kinetics of sc-to-tc conversion in R275G/S mutants are warranted. Indeed, activation of Glu-plasminogen and Lys-plasminogen varies with the tPA form, and tPA activation is influenced by the plasmin form. In addition, the assembly of the ternary tPA-fibrin(ogen)-plasmin(ogen) complex depends on the specific molecular forms of tPA, plasmin(ogen), and fibrin(ogen) [26], [43].

An important remaining question is whether endogenous tc-tPA cleaved at K277 can be detected in human plasma under physiological conditions. More broadly, whether such a form arises in specific microenvironments or under pathological proteolytic imbalances, such as in cancer or Alzheimer’s disease [44], [45], remains unknown.

Previous studies have reported conflicting results. Early work using tPA-R275X mutants (substitutions other than Arg or Lys) did not detect tc(K277)-tPA after incubation with plasmin or fibrin clots [36], [46], whereas another study reported cleavage at K277 after treatment with plasmin-Sepharose [24]. These discrepancies suggest that detection of tc(K277)-tPA may depend on experimental conditions, including materials, methods, and analytical approaches, and therefore remains unresolved.

Moreover, mutations at position I276 have been shown to promote tc(K277)-tPA generation [47], possibly by altering plasmin’s specificity at the primary cleavage site. Although R275 is highly conserved across species, I276 shows some variability. In humans, the I276L variant is reported in UniProt, but no clinical significance has been associated with it to date.

Further studies are required to clarify the determinants governing differential cleavage at R275 vs K277 and to better understand the kinetics and conditions that may favor each processing event.

Currently approved thrombolytics, such as recombinant alteplase, contain a mixture of sc-tPA and tc-tPA, with sc-tPA comprising at least 60% [6]. Although no long-term clinical differences have been definitively linked to sc-tPA/tc-tPA ratios, a lower sc-tPA/(sc-tPA+tc-tPA) ratio has been associated with an increased risk of symptomatic intracerebral hemorrhage and early seizures, potentially through modulation of N-methyl-D-aspartate receptor signaling [6].

*In vivo* studies are therefore required to determine whether R275 mutants differ from existing tPA preparations in efficacy or safety in stroke models. The functional divergence observed between sc-tPA and the 2 tc-tPA forms examined in this study suggests possible avenues for therapeutic optimization, but translation will require pharmacokinetic, pharmacodynamic, safety, and efficacy studies. TNK, a tPA mutant engineered for increased resistance to PAI-1 and enhanced fibrin affinity [29], [48], already allows bolus-only administration, unlike the bolus-plus-infusion regimen required for alteplase. Because TNK also contains R275 and K277, corresponding variants could be generated and tested experimentally.

In conclusion, our findings support Lys277 as an alternative cleavage site that can generate a distinct tc-tPA with altered fibrinolytic properties *in vitro* and *ex vivo*. These results refine the current understanding of tPA chain-form heterogeneity and provide a rationale for further mechanistic and preclinical studies of Lys277-dependent tPA processing.
