# Supplementary material for: Alternative Lys277 cleavage modulates tissue plasminogen activator processing and fibrinolysis
Source: Res Pract Thromb Haemost. 2026 Oct 12;10(7):107008. doi: 10.1016/j.rpth.2026.107008 (PMC13756511; doi:10.1016/j.rpth.2026.107008)
Supplement: Supplementary file 1 [file mmc1.docx]

**Supplemental Materials**

**Alternative Lys277 cleavage modulates tissue plasminogen activator processing and fibrinolysis**

Simon Lebatard,^1^ Laurent Lebouvier,^1^ Yannick Hommet,^1^ Anne Ciavatti Belpois,^1^ Sacha Ahuir,^1^ Viviane A. Bastos,^2^ Solenne Chardonnet,^2^ Donna Pinheiro,^2^ Robert L Medcalf,^3^ Sara Martinez de Lizarrondo,^1^ Richard Macrez,^1,4^ and Denis Vivien^1,5^

^1^University of Caen Normandie, Inserm, Normandie University, PhIND UMR-S 1237, BB@C Institute, 14000 Caen, France; ^2^Sorbonne Université, US PASS, Plateforme Post-Génomique de la Pitié-Salpêtrière (P3S), Paris, France; ^3^Molecular Neurotrauma and Haemostasis Laboratory, Australian Centre of Blood Diseases, School of Translational Medicine, Monash University, Melbourne, Victoria, Australia; ^4^Departement of Emergency medicine, Caen-Normandie University Hospital, CHU, Caen, France and ^5^Department of Clinical Research, Caen-Normandie University Hospital, CHU, Caen, France

# Supplemental Methods

## Protein modelling

P00750-1 (from UniProt) amino acid sequence (from amino acids S1-P527) of human tPA was used for 3D protein structure predictions. Predictions were executed (01/23/2025) using AlphaFold (version 2) with a Google Colab notebook (alphafold21_predict_colab.ipynb) with default settings. Best model according to the Colab notebook was used to render and export with ChimeraX (version 1.6.1). 3D protein was represented as cartoon backbone.

**Production of recombinant human tPA mutants**

### Site-directed mutagenesis and plasmids constructs

The human tPA cDNA sequence, containing a 6xHis-tag followed by -Gly-Ser- between Arg-1 and Ser1, was amplified by polymerase chain reaction with the following primers: 5′-TTTGGATCCTCTTACCAAGTGATCT-3′ and 5′-TTTCTCGAGTCACGGTCGCATGTTG-3′ (with a His6 tag at the N-terminal position). Polymerase chain reaction products were inserted into a pcDNA3.1 vector (Invitrogen). Mutagenesis of wildtype tPA (WT) was performed with the Q5 site-directed mutagenesis kit (New England Biolabs). tPA mutations were obtained with the following primers: R275G with 5’-CCTCAGTTTGGCATCAAAGGAG-3’ and 5’-TGGCTGTACTGTCTCAGG-3’, R275S with 5’-GCCTCAGTTTAGCATCAAAGGAG-3’ and 5’-TGGCTGTACTGTCTCAGG-3’ and, K277S with 5’-GTTTCGCATCAGCGGAGGGCTCTTCG-3’ and 5’-TGAGGCTGGCTGTACTGT-3’. Mutations were confirmed by sequence analysis.

**Protein production**

Recombinant proteins were produced using HEK-293T cell line (passage < 20, CRL-3216, ATCC). Petri dishes (10cm of diameter) were coated with 2% ECM (E1270, Sigma-Aldrich) diluted in DMEM high glucose (D5671, Sigma-Aldrich) at least 6h at 37°C. Cells were seeded with DMEM-GlutaMAX (32430027, Gibco) supplemented by 10% of FBS (FB-1001/500, Biosera) and maintained in a CO_2_ incubator (5%CO_2_, 37°C, humidified). Cells (<70% confluency) were transiently transfected overnight with calcium-phosphate and plasmids (8µg DNA / dish) using HEPES (51558, Sigma-Aldrich) and water (46-000-CI, Corning). Medium was then replaced by DMEM high glucose supplemented by 1mM of sodium pyruvate (11360039, Gibco), 1mM of L-glutamine (25030024, Gibco) and 3mg/L of aprotinin (A3428, Sigma-Aldrich). Medium were harvested after 4 to 5 days and centrifuged during 20min at 5000 rpm at 4°C to remove cells. Supernatants were stored at -20°C until purification.

**Protein purification and quantification**

Recombinant proteins, used *in vitro*, were purified with a Ni-NTA column placed in an ÄKTA prime plus. After thawing, supernatants with proteins were filtered with a Durapore 0.45µm PVDF membrane. Proteins were concentrated with a Pierce™ Crossflow Filtration Cassette (MWCO) on ice and mixed after with a binding buffer (20mM NaPO_4_H_2_, 500mM NaCl, 5mM imidazole, pH = 8.0). Proteins were washed with a washing buffer (20mM NaPO_4_H_2_, 500mM NaCl, 10mM imidazole, pH = 8.0) and eluted with an elution buffer (20mM NaPO_4_H_2_, 500mM NaCl, 750mM imidazole, pH = 8.0) at room temperature. Elution fractions were frozen at -20°C and analysed by a stain-free gel SDS-PAGE. Selected elution fractions were pooled and dialysed 2 times in 2 days in 2L 0.3M HEPES buffer (pH=7.40) at 4°C with a Spectra/Por membrane (MWCO 10-12kDa). After dialysis, purified recombinant proteins were aliquoted and stored at -20°C. All proteins concentrations were determined with Coomassie blue SDS-PAGE. The final tPA variants contain the 6xHis-tag between Arg35 and Ser36.

**Electrophoresis and Coomassie staining**

For purified proteins quantification, a vial of alteplase (Actilyse®, Boehringer Ingelheim) was diluted in ultrapure water to serve as protein standard. Concentration of diluted alteplase was verified with a microvolume spectrophotometer (N50, Implen) with 65000Da as molecular weight (taking glycosylation into account) and 109385M^-1^.cm^-1^ as extinction coefficient (from ProtParam, Expasy, using P00750-1 amino acid sequence from Ser1-Pro527). Proteins were heated at 95°C for 5min in a reducing sample buffer (50% (v/v) glycerol, 6% (w/v) SDS, 12.5% (v/v) β-mercaptoethanol (βME), 0.2% (w/v) bromophenol blue, pH = 8.30). Purified proteins and alteplase as standard were resolved simultaneously by reducing (βME) SDS-PAGE using 12% polyacrylamide gels (4568043/4568046, BioRad) in a running buffer (25mM Tris, 192 mM glycine, 0.1% SDS) at constant voltage (200V). Proteins in gels were stained with Coomassie blue (1 tab / 400mL Coomassie (B4921), 40% methanol, 10% acetic acid) for 2-3h at room temperature under stirring. Gels were distained (40% methanol, 10% acetic acid), rehydrated in ultrapure water and acquired on the ChemiDoc MP Imaging System (BioRad). Purified proteins quantification was estimated by densitometric analysis with ImageLab software (BioRad). Protein band intensities were quantified by densitometric analysis using ImageLab software (Bio-Rad) using the adjusted volume (band volume corrected for local background). Calibration curves plotting adjusted volume against alteplase amounts displayed robust linearity (R^2^ > 0.99; Supplementary Figure 2). Final variant concentrations were interpolated strictly within this linear range; whenever a sample signal exceeded the standard curve range, the preparation was systematically diluted and re-analyzed to avoid signal saturation.

Forms (sc-tPA/tc-tPA) of purified tPA variants were estimated in the same way as above with reducing (βME) SDS-PAGE using 10% polyacrylamide gels (4568033, BioRad). A protein ladder (1610363, BioRad) was used to estimated molecular weight.

**Western blot**

Resistance to plasmin cleavage was assessed by Western blot analysis. Purified tPA variants (1538nM final concentration; 1µg loaded per lane) were incubated with increasing amounts of human plasmin (HPlasmin, ERL) as indicated under each lane: 0, 0.0025, 0.005, 0.01, 0.02, 0.025, 0.04, 0.1, 0.2, and 1µg (corresponding to final reaction concentrations of 0, 3, 6, 12, 24, 30, 48, 120, 241, and 1205nM, respectively) for 1h at 37°C in a dry oven after gently mixing with pipette. The reaction was stopped by the addition of reducing sample buffer and heated at 95°C for 5min. Proteins were resolved by SDS-PAGE using 10% or 12% gels (4568033/4568043, BioRad) in a running buffer (25 mM Tris, 192 mM glycine, 0.1% SDS) at constant voltage (200V). Migration was verified with a stain-free gel acquisition on the ChemiDoc. Gel proteins were transferred onto a low fluorescent PVDF membrane (IPFL00010, Millipore) with the Trans-Blot Turbo Transfer System (Mixed MW, 7min) using the trans-blot turbo transfer solution (10026938, BioRad). Transfer efficiency was verified by stain-free blot acquisition on the ChemiDoc. Membranes were blocked with PBST (137mM sodium chloride, 2.7mM potassium chloride, 10mM sodium phosphate dibasic, 1.8mM potassium phosphate monobasic, 1% (v/v) polysorbate 20) supplemented with 1% (w/v) BSA (A9647, Sigma-Aldrich) for 1h at room temperature under gentle agitation. Then, membranes were incubated overnight at 4°C with an antibody against the tPA (1/1600, ab62763, Abcam) on a tube roller. Membranes were rinsed 3 times 10min with PBST. Membranes were incubated with HRP-conjugated secondary antibodies directed against IgG of the corresponding species (1/50000, A0545, Sigma-Aldrich) for 1.5h at room temperature under stirring protected from light. Membranes were rinsed 3 times 10min with PBST and 1 time for 5min in ultrapure water. Proteins bands were revealed with chemiluminescent reagents (3mL, 1705060, BioRad) on the ChemiDoc after an incubation of 3-5min protected from light. Proportion of sc-tPA and tc-tPA was estimated by densitometric analysis with ImageLab software (BioRad). A molecular weight marker (1610373 or 1610374, BioRad) was used and detected by Cy5 acquisition on the ChemiDoc. For representative acquisitions, chemiluminescent and Cy5 acquisitions were merged using ImageLab software.

Resistance to tPA cleavage in plasma was assessed by Western blot analysis as described above. Briefly, purified tPA variants (0.5µM) were incubated with citrated human platelet-poor plasma (same plasma as that used in the Plasma-based clot formation and lysis assay in the Materials and Methods) for different time points (3-60min) at 37°C in a dry oven, following gentle mixing by pipetting. Reactions were stopped by addition of reducing sample buffer and heated at 95°C for 10min. Proteins (2µL/well) were resolved by SDS-PAGE using 12% gels (4568046, BioRad) at constant voltage (100V). Gel proteins were transferred onto a low fluorescent PVDF membrane with the Trans-Blot Turbo Transfer System (Mixed MW, 7min) using the trans-blot turbo transfer solution. Transfer efficiency was verified by stain-free blot acquisition on the ChemiDoc. Membranes were blocked with PBST supplemented with 1% (w/v) BSA for 1h at room temperature under gentle agitation, then incubated overnight at 4°C with an antibody against the tPA heavy chain (1:500, IMSAHUTPAHC2A153C1MG, Innovative Research) on a tube roller. Membranes were rinsed 3 times 10min with PBST and incubated with HRP-conjugated secondary antibodies directed against IgG of the corresponding species (1/50000, A9917, Sigma-Aldrich) for 1.5h at room temperature under stirring protected from light. Membranes were rinsed 3 times 10min with PBST and 1 time for 5min in ultrapure water. Proteins bands were revealed with chemiluminescent reagents (3mL) on the ChemiDoc after an incubation of 3-5min protected from light. A molecular weight marker (1610373, BioRad) was used and detected by Cy5 acquisition on the ChemiDoc. For representative acquisitions, chemiluminescent and Cy5 acquisitions were merged using ImageLab software. Human plasmin (0.5µM) was used as a control to verify the specificity of the tPA heavy chain antibody.

**Edman sequencing**

Cleavage sites were identified by Edman sequencing. Same amount (44µg) of purified tPA variants (sc-WT, R275S and R275S-K277S) were incubated with 5 times less hu-plasmin (8.8µg) at 37°C for 1h in a dry oven. Proteins were separated in a reducing SDS-PAGE and stained with Coomassie blue as described above. Gel bands between 25-45kDa were excised and incubated at room temperature to extract the protein. After overnight incubation under shaking, the protein in solution were fixed on a PVDF disc by using a ProSorb Filter (Applied Biosystems). The PVDF disc was then introduced into an Applied Biosystems 494 automated protein sequencer (Applied Biosystems) and 11 cycles of Edman degradation were carried. The resulting amino acid sequences were matched to the sequences of public protein databases with PATTINPROT (PRABI Lyon-Gerland) and with the blastp algorithm (NCBI) against human genome.

**Conversion of sc-tPA into tc-tPA**

Conversion of sc-tPA into tc-tPA was made with plasmin coupled beads. CNBr-activated Sepharose 4B (36mg, 17043001, Cytiva) were coupled to human Glu-plasminogen (1mg, HPg2001, ERL) in part according to manufacturer instructions. Sepharose beads were rinsed 15min in an acidic buffer (1mM HCl, pH 3.7). Glu-plasminogen was dissolved in 400µL of coupling buffer (0.1M NaHCO_3_ (55761, Sigma-Aldrich), 0.5M NaCl (447300010, Thermo Scientific Chemicals), pH=8.3) and then incubated with sepharose beads in a protein LoBind tube 1.5mL (Eppendorf, 0030108116) on a tube rotator at 4°C overnight. Glu-plasminogen/beads (120µL) were incubated with 2mL of blocking buffer (0.1M Tris-HCl (T6791, Sigma-Aldrich), pH=8.0) on a tube rotator at room temperature for 2h. Glu-plasminogen/beads were rinsed 3 times alternatively with acetate wash buffer (0.1M sodium acetate (S8750, Sigma-Aldrich), 0.5M NaCl, pH=4.0) and tris wash buffer (0.1M Tris-HCl, 0.5M NaC, pH=8.0). Glu-plasminogen/beads were stored at 4°C in the storage buffer (50mM NaH_2_PO_4_ (S8282, Sigma-Aldrich), 80mM NaCl, 0.01% (w/v) sodium azide and 25% (v/v) glycerol, pH=7.4). Prior to convert Glu-plasminogen into plasmin, 9mg of alteplase (Actilyse®, Boehringer Ingelheim) were dialysed in a dialysis membrane (12-14kDa, 132700, Spectrum Labs) into 2L of 0.3M HEPES (H3375, Sigma-Aldrich) pH=7.4 under low stirring at 4°C for 48h with a change of the buffer after 24h. Then, Glu-plasminogen/beads were rinsed 3 times with phosphate buffer (50mM NaH_2_PO_4_, 80mM NaCl, pH=7.4). Dialysed alteplase was incubated with Glu-plasminogen/beads in a 15mL tube on a tube rotator at room temperature protected from light for 15h. Cleavage of alteplase and absence of free plasmin(ogen) before and after the incubation were verified with a reducing SDS-PAGE stained with Coomassie blue as described above. Then, plasmin/beads were rinsed 3 times with phosphate buffer and kept into the storage buffer at 4°C.

Same procedure as for cleavage of alteplase was performed for the conversion of WT, R275G and R275S tPA. To recover the maximum of tPAs, plasmin/beads with tPA were passed through a column (29922, Thermo Scientific).

## Enzymatic activity

### Amidolytic activity

Purified tPA variants (2µg/mL (≈30.8nM)) were mixed with Pefafluor tPA (PF tPA, CH_3_SO_2_-D-Phe-Gly-Arg-AMC⋅AcOH, 091-06, Interchim Bioscience) at different concentrations (0 to 500µM) diluted in a final volume of 110µL with buffer (100mM HEPES, 154mM NaCl, pH=7.4) in a 96-well black flat bottom plate (3925, Corning). Fluorescence intensity was measured every 30s at 440nm (excitation at 342nm) in kinetics with a fluorescent plate reader (Spark, Tecan) at 37°C for 2h. Concentrations of free AMC (cleaved Pefafluor tPA) were estimated with a standard AMC (A9891, Sigma-Aldrich) reconstituted in DMSO (D8418, Sigma-Aldrich) and diluted in the same buffer as the reaction. Standard curves were constructed on MyCurveFit.com with a four-parameter logistic regression. To eliminate plate insertion, mixing, and thermal equilibration artifacts, the initial 2-3 measurement cycles were discarded. Initial velocities (v_0_, expressed in µM/min) were determined with Excel (version 2021) from the linear regression slope across measurement cycles 4 to 9 (R^2^ > 0.98), ensuring that total substrate consumption remained below 5%. Apparent kinetic parameters (K_m_ and k_cat_) were determined by direct non-linear regression fitting to the Michaelis-Menten equation (k_cat_ model) using GraphPad Prism software (version 10.3.0). Catalytic efficiencies (k_cat_/K_m_) were computed accordingly. Each reaction was performed in duplicate; the mean was taken to represent one independent experiment. All kinetic data are presented as mean ± standard deviation (SD) across independent experiments.

Same procedure was applied with the addition of human PAI-1 (HY-P71077, MCE). Prior to the reaction, tPAs (2µg/mL (≈30.8nM)) and PAI-1 (2µg/mL (≈41.7nM)) were mixed together at 37°C for 10min in a dry oven. Each reaction was performed in singlicate. A fold reduction ratio was calculated with the k_cat_/K_m_ value without PAI-1 divided by the k_cat_/K_m_ value with PAI-1.

### Plasminogen-fibrin overlay zymography

Agarose gels were prepared at 39°C with 2% agarose low melting (A4018, Sigma-Aldrich), 20mg of non-plasminogen-depleted bovine fibrinogen (with trace amounts of plasminogen, Bovogen Biologicals) and 2.25IU of thrombin in a 23mL buffer (10mM HEPES, 150mM NaCl, 2mM CaCl_2_, 0.01% sodium azide, pH=7.4) placed in a clear square Petri dish. In parallel, a SDS-PAGE was performed at 4°C with purified tPA variants (0.2µg/lane) using 10% polyacrylamide gels (4568033, BioRad) with refrigerated running buffer. A stain free gel was done to confirm the presence of proteins. The order of proteins in wells varied with each gel to prevent the position influence. After migration, acrylamide gels were incubated 2 times at 4°C with stirring for 1h with a change every 30min in a refrigerated 2.5% Triton X-100 solution (pure water) to remove SDS. Gels were rinsed 3 times 10min at 4°C with stirring in refrigerated pure water to remove Triton X-100. Then, gels were placed on an agarose gel and left 20min at room temperature. Square Petri dishes were sealed and stored overnight at 4°C. The day after, square Petri dishes were incubated in a dry oven at 37°C up to 80min. An image was captured every 20min with an ImageScanner III (General Electrics) device using the LabScan software (General Electrics) with the same calibration. Densitometric and morphometric analyses were conducted under identical contrast and brightness parameters. Lysis areas (mm^2^) were determined using Image Lab software (Bio-Rad) by defining a freehand region of interest around each clearing halo for each incubation time. The lysis velocity (mm^2^/min) was then calculated from the slope of the linear regression curve of the lysis area plotted against incubation time. Lysis zone intensity was quantified with the halo intensity at 60min using ImageJ software (NIH). All quantitative parameters were normalized against recombinant single-chain wild-type tPA (sc-WT tPA) loaded on the same gel to correct for inter-assay and gel-to-gel variations.

# Supplemental Figures

**Supplementary Figure S1. Evolutionary conservation of the cleavage site among tPA paralogues. (A)** Consensus sequence of close tPA paralogues from Gnathostomata. uPA: urokinase-type plasminogen activator, HGFA: hepatocyte growth factor activator, and HABP2: hyaluronan binding protein 2. **(B)** Sequences of close tPA paralogues with a mutation on the P1 site other than an arginine and a lysine. Human sequences are represented in light grey.

**Supplementary Figure SS2. Quantification and linearity of recombinant tPA (alteplase) by densitometric Coomassie staining. (A)** Representative reducing SDS-PAGE (12% polyacrylamide) showing a serial dilution of clinical-grade alteplase used as a calibration standard (ranging from 0.83 to 6.67µg per lane), stained with Coomassie blue. Molecular weight markers are indicated on the left in kDa. kDa: kilo Dalton, MW: molecular weight. **(B)** Corresponding linear calibration curve plotting the densitometric adjusted volume (background-subtracted band intensity, determined using Image Lab software) against the loaded alteplase concentration. Linear regression analysis confirms strict linearity within the dynamic measurement range (R^2^ = 0.9939). Unknown recombinant tPA samples were quantified within this linear interval, with samples systematically diluted if their initial densitometric signal exceeded the upper limit of quantification. A.U.: arbitrary unit.

**Supplementary Figure SS3. Forms of tPA variants.** Upper part: representative reducing SDS-PAGE followed by Coomassie blue staining of tPA variants including two-chain single mutants and two-chain WT (from Figure 2B). Molecular weight markers are indicated on the left in kDa. kDa: kilo Dalton, MW: molecular weight. Lower part: proportion of sc-tPA and tc-tPA of tPA variants. Data represent the mean ± SD of 3 independent experiments for tc-WT, tc-R275G, tc-R275S and 5 independent experiments for others variants (from Figure 2B).

**Supplementary Figure S4. Kinetic profiles of tPA variants in PF-tPA hydrolysis.** Michaelis-Menten curves obtained from initial velocity of hydrolysis of PF-tPA at increasing concentrations with tPA variants (2µg/mL (≈30.8nM)) with and without PAI-1 (2µg/mL (≈41.7nM)) according to PF-tPA initial concentration. Data represents mean curves with mean v_0_ of 6 independent experiments. v_0_: initial velocity, and PF-tPA: pefafluor tPA.

**Supplementary Figure SS5.** **Fibrinolytic Effects of sc-tPA Variants Assessed by ROTEM. (A)** ROTEM profiles of clots firmness of whole blood mixed with r ex-tem/star-tem and sc-tPA variants at 0, 4.6, or 6.9nM. Each curve represents an individual donor. Experiments were performed 4 times for each tPA variant. (**B)** ROTEM parameters obtained from ROTEM curves (A) with sc-tPA variants at 0, 4.6, or 6.9nM. Data represent the mean ± SD of 4 independent experiments for each tPA variant. Comparisons were performed using the Kruskal-Wallis test followed by Dunn’s post hoc multiple-comparison test, with the sc-WT condition as the reference group, except for conditions with n<3. ML: maximum lysis, LOT: lysis onset time, LT: lysis time, FS: fibrinolysis speed, and FSc: corrected fibrinolysis speed.

**Supplementary Figure S6. Conversion of sc-R275S into the two-chain form in human plasma.** **(A)** Western blot analysis of tPA variants (0.5 µM) incubated with or without human plasma at 37 °C for 0 to 60 min. Lower panels show a higher-contrast view of the dotted cropped area. Cleaved tPA corresponds to the tPA heavy chain generated after proteolytic conversion of sc-tPA. Protein molecular weight standards were loaded on both sides of the gel (lanes 1 and 15). **(B)** Western blot analysis demonstrating the specificity of the tPA heavy chain antibody. The antibody selectively recognizes the ~35 kDa heavy chain generated upon cleavage of tPA, with no detectable nonspecific bands. IT: incubation time, kDa: kilo Dalton, MW: molecular weight.

**Supplementary Figure S7. Fibrinolytic Effects of tc-tPA Variants Assessed by ROTEM. (A)** ROTEM profiles of whole-blood clot firmness with r ex-tem/star-tem and tc-tPA variants at 0, 4.6, or 6.9nM. Each curve represents an individual donor. Experiments were performed 4 times for each tPA variant. **(B)** ROTEM parameters derived from the ROTEM curves (A) for tc-tPA variants at 0, 4.6, or 6.9nM. Data represent mean ± SD of 4 independent experiments per tPA variant. Comparisons were performed using the Kruskal-Wallis test followed by Dunn’s post hoc multiple-comparison test, with sc-WT as the reference group, except for conditions with n<3. Significance is indicated as *p<0.05. ML: maximum lysis; LOT: lysis onset time; LT: lysis time; FS: fibrinolysis speed; FSc: corrected fibrinolysis speed.
